# Differential expression of CCN-family members in primary human bone marrow-derived mesenchymal stem cells during osteogenic, chondrogenic and adipogenic differentiation

**DOI:** 10.1186/1478-811X-3-5

**Published:** 2005-03-17

**Authors:** Norbert Schutze, Ulrich Noth, Jutta Schneidereit, Christian Hendrich, Franz Jakob

**Affiliations:** 1 Orthopaedic University Hospital, Molecular Orthopaedics, Brettreichstrasse 11, 97074 Würzburg, Germany

## Abstract

**Background:**

The human cysteine rich protein 61 (CYR61, CCN1) as well as the other members of the CCN family of genes play important roles in cellular processes such as proliferation, adhesion, migration and survival. These cellular events are of special importance within the complex cellular interactions ongoing in bone remodeling. Previously, we analyzed the role of CYR61/CCN1 as an extracellular signaling molecule in human osteoblasts. Since mesenchymal stem cells of bone marrow are important progenitors for various differentiation pathways in bone and possess increasing potential for regenerative medicine, here we aimed to analyze the expression of CCN family members in bone marrow-derived human mesenchymal stem cells and along the osteogenic, the adipogenic and the chondrogenic differentiation.

**Results:**

Primary cultures of human mesenchymal stem cells were obtained from the femoral head of patients undergoing total hip arthroplasty. Differentiation into adipocytes and osteoblasts was done in monolayer culture, differentiation into chondrocytes was induced in high density cell pellet cultures. For either pathway, established differentiation markers and CCN-members were analyzed at the mRNA level by RT-PCR and the CYR61/CCN1 protein was analyzed by immunocytochemistry.

RT-PCR and histochemical analysis revealed the appropriate phenotype of differentiated cells (Alizarin-red S, Oil Red O, Alcian blue, alkaline phosphatase; osteocalcin, collagen types I, II, IX, X, cbfa1, PPARγ, aggrecan). Mesenchymal stem cells expressed CYR61/CCN1, CTGF/CCN2, CTGF-L/WISP2/CCN5 and WISP3/CCN6. The CYR61/CCN1 expression decreased markedly during osteogenic differentiation, adipogenic differentiation and chondrogenic differentiation. These results were confirmed by immuncytochemical analyses. WISP2/CCN5 RNA expression declined during adipogenic differentiation and WISP3/CCN6 RNA expression was markedly reduced in chondrogenic differentiation.

**Conclusion:**

The decrease in CYR61/CCN1 expression during the differentiation pathways of mesenchymal stem cells into osteoblasts, adipocytes and chondrocytes suggests a specific role of CYR61/CCN1 for maintenance of the stem cell phenotype. The differential expression of CTGF/CCN2, WISP2/CCN5, WISP3/CCN6 and mainly CYR61/CCN1 indicates, that these members of the CCN-family might be important regulators for bone marrow-derived mesenchymal stem cells in the regulation of proliferation and initiation of specific differentiation pathways.

## Background

Members of the cysteine-rich61/connective tissue growth factor/nephroblastoma overexpressed (CCN 1–3) family of genes (CCN-family) function in processes such as proliferation, differentiation as well as cell adhesion, migration and survival [1-3]. Additional members of this family are Elm1/WISP1/CCN4, rCop1/WISP2/CTGF-L/CCN5 and WISP3/CCN6 [1,2,4]. The proteins mainly represent matrix associated signal molecules and share a common modular structure [5,6]. N-terminal amino acids have been shown to be important for secretion of CYR61/CCN1 [7]. Although CCN proteins share an insulin-like growth factor binding protein (IGFBP)-like motif, no clear experimental significance exists to suggest a function in the IGF signaling pathway [8]. The von Willebrand type C domain (VWC), the trombospondin type I domain and the C-terminal module (which is absent in CTGF-L/WISP2) are considered to be important for protein-protein interactions, either oligomerisation (VWC) or interactions with extracellular matrix molecules and receptors. Interaction partners of CCN proteins include integrin receptors [9-15], surface heparan sulfate proteoglycans (CYR61) [11], decorin and biglycan (WISP1) [16], and fibulin 1C (NOVH) [17]. Additional binding partners are likely to exist since interactions of CCN proteins in additional signal transduction pathways such as BMP and TGF-β signaling for CTGF/CCN2 [18] and CYR61/CCN1, intracellular calcium signaling (NOV/CCN3) [19], the notch pathway (NOV) [20] and the Wnt pathway (CYR61/CCN1 have been described [21].

An important target for CCN proteins could be bone and cartilage since the expression of CCN members in chondrocytes and osteoblasts is known from animal models and human tissues, and the majority of the above mentioned receptors and pathways are also relevant to skeletal homeostasis. CYR61/CCN1 is involved in chondrogenesis in mice [22], is expressed in human bone at sites of bone remodeling, in hypertrophic chondrocytes at the growth plate [23], and in the fracture callus in rats [24]. In human osteoblasts CYR61/CCN1 expression is regulated by a variety of bone-relevant growth factors [25]. CTGF/CCN2 is also expressed in bone and cartilage [8,26]. The role in skeleletal homeostasis and cartilage development is strengthened by a mouse model with functional inactivation [27]. CCN5 has been implicated in osteoblast and chondrocyte function [28]. CCN3 also is expressed in chondrocytes and could play a role in chondrocyte differentiation [3,6]. Mutations in the CCN6 gene are known to be associated with pseudorheumatoid dysplasia [29]. Therefore, CCN proteins are relevant for skeletal growth and development, e. g. bone and cartilage formation and function and some of the CCN proteins could as well be important in fracture repair and bone remodeling.

Bone marrow-derived mesenchymal stem cells (MSC) are versatile cells which can differentiate into various cell types including osteoblasts, chondrocytes and adipocytes. MSC express markers of additional cell types, depending on the environment or cell culture conditions [30-36]. Therefore, this *in vitro *differentiation system of osteogenic, adipogenic and chondrogenic differentiation allows to investigate for genes which are temporally expressed during differentiation pathways. Here we investigated the expression patterns of CCN family members during these differentiation pathways using human bone marrow-derived MSC. The results indicate that the expression of CCN-family members is dependent on the differentiation status of human MSC *in vitro*.

## Results

### Lineage-specific differentiation of bone marrow-derived MSC

**Figure 1:**
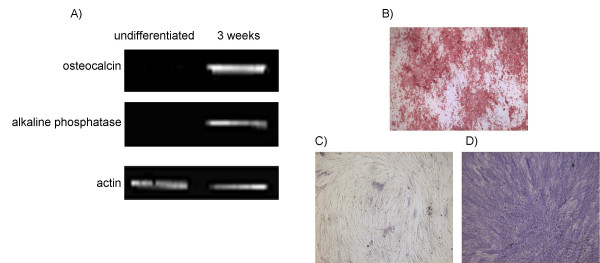
**Marker analysis of osteoblasts derived from MSC**. Human bone marrow-derived MSC were differentiated into osteoblasts according to the methods section. were compared A) RT-PCR results for alkaline phosphatase and osteocalcin, B) Alizarin Red staining of calcium phosphate deposits in differentiated osteoblasts, C and D) Alkaline phosphatase staining of MSC and osteoblasts after 4 weeks of differentiation, respectively, (scale bar = 100 μm).

**Figure 2:**
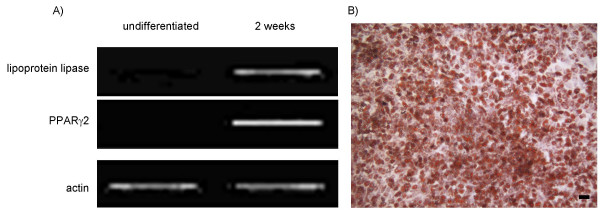
**Marker analysis of adipocytes derived from MSC**. Human bone marrow-derived MSC were differentiated into adipocytes according to the methods section. A) RT-PCR results for lipoprotein lipase (LPL) and peroxisome proliferators-activated receptor γ2 (PPARγ2), B) staining for lipid droplets (Oil-Red O), (scale bar = 100 μm).

**Figure 3:**
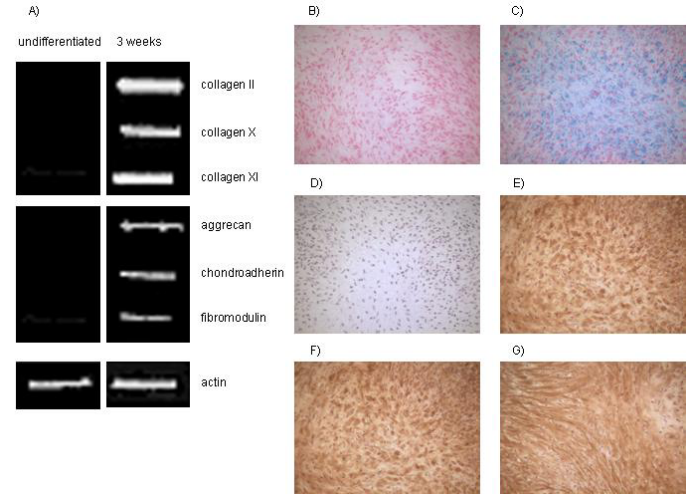
**Marker analysis of chondrocytes derived from MSC**. Human bone marrow-derived MSC were differentiated into chondrocytes according to the methods section. A) RT-PCR analyses for chondrogenic markes as is indicated, B) H & E-staining, C) Alcian blue staining (H & E-counterstaining, D) example of negative control for immunohistochemical analyses (DAPI-counterstained), E) immunohiostochemical staining for collagen type II, F) immunohiostochemical staining for chondroitin sulphate 4, G) immunohiostochemical staining for chondroitin sulphate 6, (scale bar = 100 μm).

MSC obtained from 4 different patients each were used throughout the individual differentiation pathways for this study. As was described previously [36] monolayer cultures treated with osteogenic supplements showed a high percentage of alkaline-positive cells, stained positive for mineralized matrix deposition and expressed the osteogenic specific marker genes alkaline phosphatase and osteocalcin (Fig. 1). Monolayer cell cultures treated with adipogenic supplements showed cytoplasmic lipid droplets and expressed LPL and PPARγ2, characteristic for adipocytes (Fig. 2). High density pellet cell cultures were composed of morpholocically distinct, chondrocyte-like cells, surrounded by a sulfated proteoglycan-rich extracellular matrix and expressed collagen type II (Fig. 3).

### Expression of CCN family members in undifferentiated MSC

**Figure 4:**
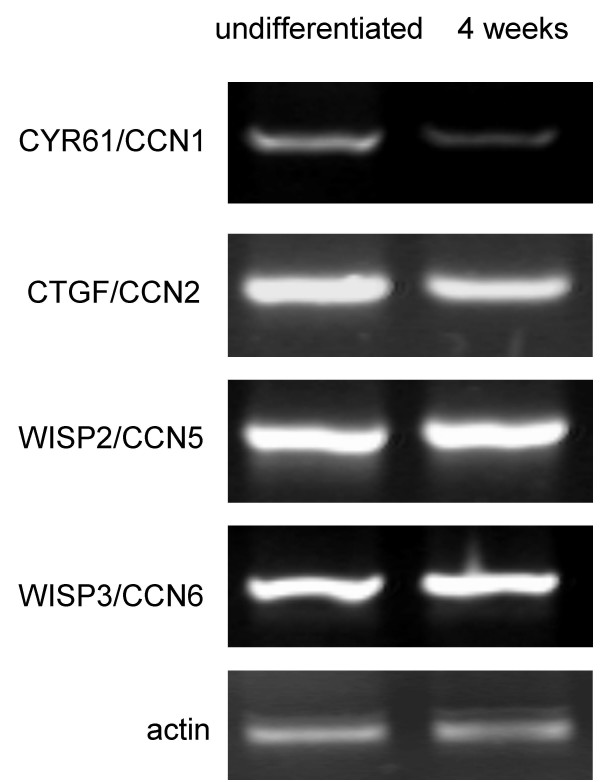
**RT-PCR analysis of CCN family members during osteogenic differentiation**. Human bone marrow-derived MSC were differentiated into osteoblasts according to the methods section. Total RNA was isolated and reverse transcribed from undifferentiated cells and differentiated cells after 4 weeks. PCR products for CCN family members as indicated were subjected to agarose gel electrophoresis.

**Figure 6:**
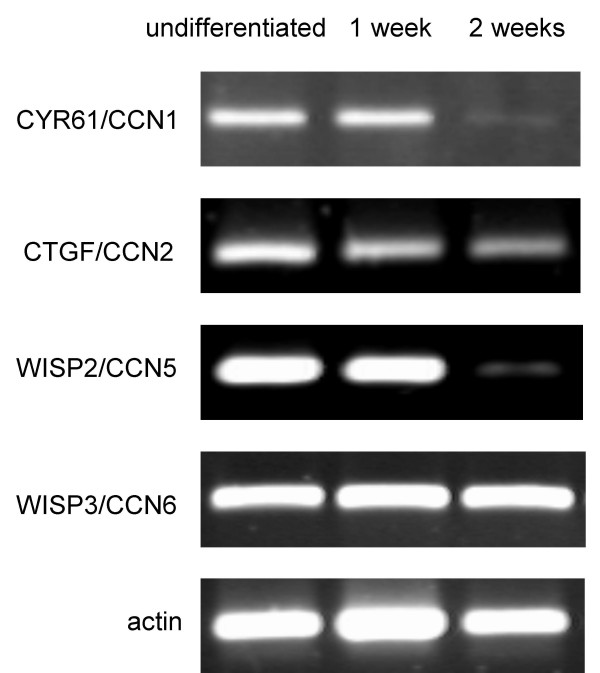
**RT-PCR analysis of CCN family members during adipogenic differentiation**. Human bone marrow-derived MSC were differentiated into adipocytes according to the methods section. Total RNA was isolated and reverse transcribed from undifferentiated cells and differentiated cells after 1 and 2 weeks. PCRproducts for CCN family members as indicated were subjected to agarose gel electrophoresis.

**Figure 7:**
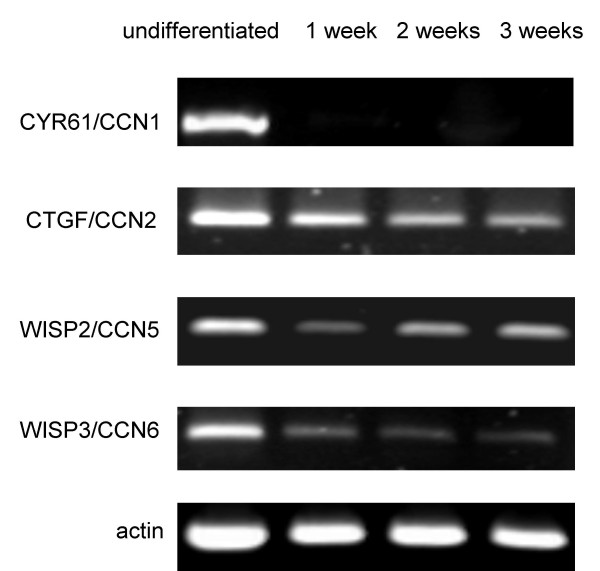
**RT-PCR analysis of CCN family members during chondrogenic differentiation**. Human bone marrow-derived MSC were differentiated into chondrocytes for 3 weeks in high-density cell pellets according to the methods section. Total RNA was isolated and reverse transcribed from undifferentiated cells and differentiated cells after 1, 2 and 3 weeks. PCR products for CCN family members as indicated were subjected to agarose gel electrophoresis.

Bone marrow-derived MSC prior to the initiation of the various differentiation pathways expressed CYR61/CCN1, CTGF/CCN2, CCN5 and CCN6 genes by RT-PCR analysis at high levels (Fig 4, 6 and 7, left lane each). No signals were obtained for CCN3 and for CCN4 expression in undifferentiated MSC as well as during differentiation pathways derived thereof, even after variations of the RT-PCR procedure. Further analyses, therefore, focused on CYR61/CCN1, CTGF/CCN2, WISP2/CCN5 and WISP3/CCN6 (see below).

### Differential expression of CYR61/CCN1 during osteogenic differentiation

**Figure 5:**
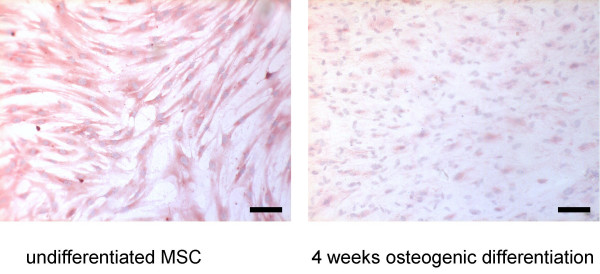
**Expression of the CYR61 protein during osteogenic differentiation**. Human bone marrow-derived MSC were differentiated into cells of the osteogenic lineage. Undifferentiated cells and differentiated cells after 4 weeks were fixed and subjected to immunohistochemistry as outlined in the methods section (scale bar = 100 μm).

A marked decrease in the expression of CYR61/CCN1 was observed during osteogenic differentiation. At the mRNA level RT-PCR analysis revealed a 6.9 ± 2.2-fold decrease (n = 4) in CYR61/CCN1 levels after 4 weeks of osteogenic differentiation, compared to undifferentiated MSC (Fig. 4). RT-PCR analysis for other CCN-members in this study did not display changes in PCR product intensity during osteogenic differentiation. At the protein level, immunocytochemistry showed a decrease in CYR61/CCN1 signal from differentiated cells compared to undifferentiated MSC (Fig. 5).

### Differential expression of CYR61/CCN1, CTGF/CCN2 and WISP2/CCN5 during adipogenic differentiation

RT-PCR analysis of the CCN family members during adipogenic differentiation is shown in Fig 6. CYR61/CCN1 and WISP2/CCN5 were expressed prior to the initiation of adipogenesis and in initial stages of the differentiation pathway. However, in differentiated adipocytes after 2 weeks of differentiation almost undetectable levels were observed (n = 4). Due to the low expression levels densitometry was not possible. RT-PCR for CTGF/CCN2 revealed a slight reduction of the PCR product levels towards differentiated adipocytes (2.1 ± 0.8-fold). WISP3/CCN6 PCR product intensity did not change during adipogenic differentiation.

### Differential expression of CYR61/CCN1 and WISP3/CCN6 during chondrogenic differentiation

**Figure 8:**
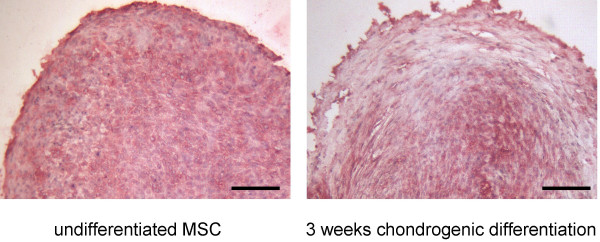
**Expression of CYR61 protein in high-density pellets during chondrogenic differentiation**. Human bone marrow-derived MSC were differentiated into chondrocytes in high-density cell pellets. Slides from pellets of the undifferentiated cells and differentiated cells after 3 weeks were cut through the center of the pellet, fixed and subjected to immunohistochemistry as outlined in the methods section. (scale bar = 100 μm).

In high-density cell pellets prior to the initiation of chondrogenic differentiation RT-PCR analysis for CYR61/CCN1 and WISP3/CCN6 revealed high levels of RNA expression. During differentiation after one week signals for CYR61/CCN1dropped to undetectable levels. (Fig. 7). Therefore, densitometry for CYR61/CCN1 RT-PCR products after differentiation was not possible. PCR product intensity for WISP3/CCN6 was reduced 5.5 ± 0.6-fold (n = 4). RT-PCR for CTGF/CCN2 revealed a slight reduction during chondrogenic differentiation of 1.9 ± 0.6-fold. WISP2/CCN5 PCR products did not reveal differences in intensity along the chondrogenic differentiation of MSC. Immunohistochemistry of slides cut through the center of the pellets indicated high levels of CYR61/CCN1 protein prior to the initiation of differentiation. After three weeks of chondrogenic differentiation a reduced signal intensity at the periphery of the pellet was observed (Fig. 8).

## Discussion

CCN proteins play important roles in growth and differentiation and are involved in cellular processes such as migration, adhesion and survival [1,2,6]. A major role for CYR61/CCN1 and CTGF/CCN2 in bone and cartilage development is indicated by animal studies [6,22,27,37,38]. In the adult both CCN-family members have been associated with remodeling and repair in tissues such as bone and cartilage, muscle, the vascular system and the nervous system as reviewed in [2,3,39,40].

The culture of human bone marrow-derived MSC applied in this study allows the analysis of CCN protein expression and function during differentiation pathways. Particularly, we investigated the expression of CCN family members during the differentiation of human bone marrow-derived MSC into osteoblasts, adipocytes and chondrocytes. Results showed that CYR61/CCN1 in all pathways, and CTGF/CCN2, WISP2/CTGF-L/CCN5 and WISP3/CCN6 in specific pathways were differentially expressed and therefore could participate in MSC function and lineage progression.

Quantitative measurements of mRNA levels have not been performed in this study. This limits the possibility to detect subtle differences in CCN mRNA expression. However, the reproducibly observed differences for CYR61/CCN1, WISP2/CCN5 and WISP3/CCN6 expression along differentiation pathways were sufficiently intense to be detected by the conventional RT-PCR procedure applied.

Except for NOV/CCN3 and WISP1/CCN4, all CCN family members investigated were expressed in undifferentiated MSC and/or along differentiation pathways derived thereof. Expression of lineage-specific cell markers at the RT-PCR level as well as histochemical analysis for mineralized matrix deposition, lipid droplets and cartilage-specific extracellular matrix revealed that the appropriate conditions for differentiation of MSC were applied. Whereas NOV/CCN3 expression has been described in the murine growth plate and murine chondrosarcomas [41], our failure to detect NOC/CCN3 along chondrogenic differentiation could rely on species and/or cell specific differences, however, we cannot exclude low expression levels below the detection limit of the conventional RT-PCR procedure.

We never observed an increase of PCR product intensity in RT-PCR of any CCN family member during lineage-specific differentiation. Therefore, corresponding steady state mRNA levels of CCN members were highest in undifferentiated MSC compared to differentiated cell types. This might indicate specific functions of CCN proteins in the precursor cells prior to the onset of differentiation pathways. These functions could be associated with the known positive growth regulation capabilities of CYR61/CCN1, CTGF/CCN2 and WISP2/CCN5.

Particularly, CYR61/CCN1 could play a significant function in MSC *in vitro *since its expression was largely decreased during all differentiation pathways under study. The decreased PCR product intensity in RT-PCR analysis during differentiation, however, does not prove the absence of particular functions in differentiated cells. The protein can be stably bound within the extracellular matrix. The results of immunohistochemistry for CYR61/CCN1 expression after osteogenic differentiation indicated lower but still detectable signals and in high-density cell pellet cultures used for chondrogenic differentiation a reduced but significant protein expression was observed. Despite lower expression of CYR61/CCN1 in differentiated cells, the mRNA and protein levels can be upregulated by various bone relevant growth factors [25]. Our finding of undetectable CYR61/CCN1 expression in MSC-derived chondroctes is in contrast to reports describing the CYR61-dependent regulation of chondrogenesis in mouse limb bud mesenchymal stem cells [22] and its expression in chondrocytes during fracture repair in rats [24]. This difference could result from species and/or cell specific differences or depend on the detection limit as is discussed above.

CTGF/CCN2 was constitutively expressed at the RT-PCR level during osteogenic differentiation and only slightly reduced during adipogenic and chondrogenic differentiation. CTGF/CCN2 is able to stimulate the expression of markers of differentiated osteoblasts and chondrocytes *in vitro *[8,26,42]. WISP2/CCN5 expression was only decreased during adipogenic differentiation in a pattern that paralleled CYR61/CCN1 expression, but was constitutively expressed in the other differentiation pathways. WISP3/CCN6 expression was downregulated during chondrogenic differentiation. Since mutations in the WISP3/CCN6 gene result in pseudorheumatoid dysplasia [29] an important role in cartilage formation and/or initiation of chondrogenesis is possible.

With regard to the molecular signalling mechanisms, the CCN family members act in a variety of signalling pathways such as notch, BMP, Wnt and intracellular calcium signalling [18-21] as is reviewed in [2,3]. Several of these identified pathways are relevant for differentiation lineages and cell fate decisions. Particularly the notch, BMP and Wnt signaling pathways are relevant for the developing skeleton. The adhesive signalling capabilities, mainly studied for CYR61/CCN1 are of special relevance for tissue homeostasis in the bone and cartilage microenvironment [11,13,43]. Mainly CYR61/CCN1 [22] and CTGF [27,44-46] appear to regulate bone and cartilage formation in embryonic development. Tissue repair in the adult could comprehense cellular processes occurring in the developmental stage. Thus, the growing importance of MSC for targeted tissue repair could, in part, rely on CCN-protein function. The differential expression of CCN family members, particularly CYR61/CCN1 mainly in MSC compared to differentiated cells could indicate a functional importance of these proteins for tissue engineering purposes.

## Conclusion

**Figure 9:**
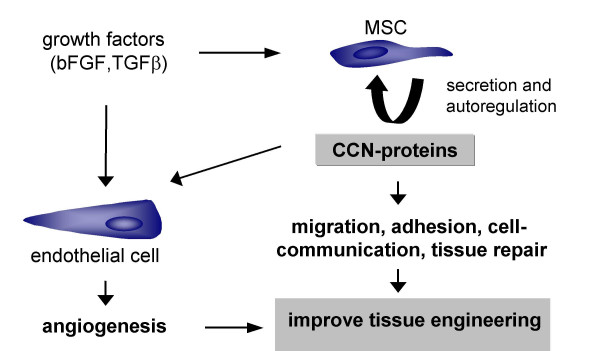
**CCN proteins could improve tissue engineering strategies**. Human bone marrow-derived MSC express and secrete CCN family proteins. Mainly CCN 1, 2, 5 and 6 are expressed in undifferentiated cells. Particularly CYR61/CCN1 and CTGF/CCN2 are important regulators in the developing skeleton and represent angiogenic modulators. Targeted tissue repair in the adult could, in part, rely on this dual role of CCN proteins allowing for improvement of tissue engineering strategies.

The high expression of CCN family members in MSC and the sharp decline in RNA expression levels during differentiation of MSC into osteoblasts, adipocytes and chondrocytes, particularly for CYR61/CCN1 but also for CTGF/CCN2, WISP2/CCN5, WISP3/CCN6 in selected pathways, could indicate that these members of the CCN-family might be important regulators for bone marrow-derived MSC. MSC possess a growing importance in targeted tissue repair in the adult. Repair processes in the adult could involve cellular events occuring during development. Since the CCN family members (mainly CYR61/CCN1 and CTGF/CCN2) play important roles in the developing skeleton and in angiogenesis, these factors likely could play a role in targeted tissue repair and could improve tissue engineering strategies (Fig. 9).

## Methods

### Materials

FCS was supplied by Gibco (Eggenstein, Germany). Taq Polymerase was purchased from Amersham Pharmacia (Freiburg, Germany). An anti mouse CYR61 polyclonal antiserum was obtained from Munin Corp. (Chicago, USA). All other chemicals were of the highest purity available.

### Isolation of MSC

MSC were isolated from human bone marrow obtained from the femoral head of patients undergoing total hip arthroplasty using a modified protocol [36] originally described by Haynesworth et al. [31]. Usage of patient material was approved by the local ethics commitee of the University of Würzburg and written consent was obtained from each patient. MSC from 4 patients were used for this study (3 male, 1 female), age between 51 and 62 years. Trabecular bone plugs were harvested from the cutting plane of the femoral head and transferred to 50 ml conical tubes containing DMEM/F12 medium (PAA, Cölbe, Germany). After vortexing and centrifugation (1000 rpm, 5 min) the pellet containing bone plugs and released cells was reconstituted in DMEM/F12 medium supplemented with 10% fetal bovine serum (FBS), antibiotics (50 I.U. penicillin/ml and 50 μg streptomycin/ml) and 50 μg/ml ascorbate (complete medium). After repeated washings, the released cells were pelleted (1000 rpm, 5 min), suspended in complete medium, plated at a density of 60 × 10^6 ^cells per 150 cm^2 ^tissue culture flask and maintained at 37°C in 5% CO_2_. Non adherent cells were removed after 2 days and subsequently the medium was changed every 2 days until the cell cultures reached confluency.

### Osteogenic differentiation of MSC

Cells were plated in 6-well plates or chamber slides in complete medium. At confluency osteogenic differentiation was initiated using the complete medium supplemented with 10 mM β-glycerophosphate and 50 μg/ml ascorbate. Medium was changed every 2 days up to 4 weeks. To monitor osteogenic differentiation RT-PCR analysis for alkaline phosphatase, osteopontin and osteocalcin was performed as well as stainings for alkaline phosphatase (Sigma, Taufkirchen, Germany, Kit No. 86) and calcium phosphate deposition (Alizarin Red S) were performed as described by Nöth et al. [36].

### Adipogenic differentiation of MSC

Cells were plated in 6-well plates or chamber slides in DMEM with 10 % FBS. At confluency, adipogenic differentiation was initiated using the same medium supplemented with 1 μM dexamethasone, 0.5 mM isobutylmethylxanthine, 1 μg/ml insulin and 100 μM indomethacin. Medium was changed every 2 days up to 2 weeks. To monitor adipogenic differentiation RT-PCR analysis for lipoprotein lipase (LPL) and peroxisome proliferator-activated receptor γ2 (PPARγ2) was performed, and a staining for lipid droplets (Oil Red O) as described [33,36].

### Chondrogenic differentiation of MSC

Cells were cultured as high-density pellet cultures in a serum-free medium as described previously [32,33]. 200, 000 cells were centrifuged for 5 min and 1000 rpm in 15 ml conical tubes and the pellets cultured for 3 weeks at 37°C in 5 % CO_2_. The chondrogenic medium consisted of DMEM supplemented with 10 ng/ml transforming growth factor β1 (Stathmann, Hamburg, Germany), 100 nM dexamethasone, 50 μg/ml ascorbic 2-phosphate, 100 μg/ml sodium pyruvate, 40 μg/ml proline and ITS-plus (consisting of 6.25 μg/ml bovine insulin, 6.25 μg/ml transferrin, 6.25 μg/ml selenous acid, 5.33 μg/ml linoleic acid and 1.25 mg/ml bovine serum albumin; Sigma, Taufkirchen, Germany). Medium was changed twice per week. To monitor chondrogenic differentiation RT-PCR analysis for aggrecan and collagen types I, II, IX and X was performed. Additionally, sections were cut through the center of the pellets and stained with alcian blue and were subjected to immunohistochemical analyses for collagen type II, chondroitin sulphate 4 and 6 as described by Nöth et al. [36].

### RNA Isolation and RT-PCR

RNA isolates were obtained from cells within the differentiation pathways as well as control isolates. Controls for monolayer differentiation (i.e. into adipocytes and osteoblasts) represent confluent resting MSC at day 0 prior to the initiation of cell differentiation. Control RNA isolates for the chondrogenic differentiation pathway were done from pellets prior to the initiation of chondrogenic differentiation at day 0. RNA isolation was performed using purification columns (Qiagen, Hilden, Germany) according to the manufacturer. Cells were rinsed with PBS, lysis buffer was added, RNA was column purified and the yield was determined by spectrophotometry. cDNA synthesis was done in a 20 μl reaction using Super Script II Reverse Transcriptase (Invitrogen, Karlsruhe, Germany) with identical amounts of RNA isolated from individual differentiation pathways of MSC (1 μg). PCR was performed using a PTC-200 thermal cycler (MJ Research) in a volume of 30 μl containing 1 μl cDNA with primers (Table 1) for CCN family members as well as the housekeeping gene actin. Primers used for cell markers are presented in Table 2. The PCR buffer consisted of 10 mM Tris (pH 8.4), 50 mM KCl, 1.5 mM MgCl_2_, 0.25 mM dNTP's, 5 pmol forward and reverse primer, and 0.5 units of Taq polymerase. Cycling was 4 min at 94°C, 25 – 40 cycles (25 cycles: actin and E1α, 30 – 37 cycles for CCN members) of 94°C for 30 s, 58 – 64°C for 1 min (depending on the primers) and 72°C for 1 min, with a final 72°C step of 5 min. Sequence analysis was performed by dye terminator sequencing of the PCR products to verify specificity using an ABI 310 Sequencer. 10 μl of PCR products were separated on a 2% agarose gel containing ethidium bromide. In cases of differential PCR product intensities densitometry was performed using an LTF densitometer and the bioprofile software (LTF, Wasserburg, Germany)

**Table 1:** Primers used for RT-PCR

gene product		primer sequence	# in database entry
CYR61/CCN1	sense	5'ACCAGTCAGGTTTACTTACG 3'	962 – 981 NM_001554
	antisense	5'TGCCTCTCACAGACACTCAT 3'	1679 – 1698 NM_001554

CTGF/CCN2	sense	5'AACACCATAGGTAGAATGTAAAGC 3'	1921 – 1944 NM_001901
	antisense	5'CTGATCAGCTATATAGAGTCACTC 3'	2130 – 2153 NM_001901

WISP2/CCN5	sense	5'CACGCATAGGCTTGTATTCAGGAAC 3'	1052 – 1075 NM_003881
	antisense	5'CACGCTGCCTGGTCTGTCTGGATC 3'	1379 – 1403 NM_003881

WIS32/CCN6	sense	5'CTGTGTTACATTCAGCCTTGCGAC 3'	846 – 869 NM_003880
	antisense	5'CTTGGTTTTACAGAATCTTGAGCTC 3'	1158 – 1182 NM_003880

actin	sense	5'CGGGAAATCGTGCGTGACAT 3'	689 – 708 NM_001101
	antisense	5'GAACTTTGGGGGATGCTCGC 3'	1381 – 1400 NM_001101

**Table 2:** Primers used for RT-PCR of markers for MSC-derived differentiation pathways

Alkaline phosphatase		
sense	5'TGGAGCTTCAGAAGCTCAACACCA 3'	368 – 391 NM_000478
antisense	5'ATCTCGTTGTCTGAGTACCAGTCC 3'	798 – 821 NM_000478
Osteocalcin		
sense	5'ATGAGAGCCCTCACACTCCTC 3'	19 – 39 NM_199173
antisense	5' GCCGTAGAAGCGCCGATAGGC 3'	292 – 312 NM_199173

Lipoprotein lipase		
sense	5'GAGATTTCTCTGTATGGCACC 3'	1261 – 1281 NM_000237
antisense	5'CTGCAAATGAGACACTTTCTC 3'	1516 – 1536 NM_000237

PPARγ2		
sense	5'GCTGTTATGGGTGAAACTCTG 3'	128 – 148 NM_015869
antisense	5'ATAAGGTGGAGATGCAGGCTC 3'	458 – 478 NM_015869

Collagen type II		
sense	5'GAACATCACCTACCACTGCAAG 3'	4318 – 4339 NM_001844
antisense	5'GCAGAGTCCTAGAGTGACTGAG 3'	4684 – 4705 NM_001844

Collagen type X		
sense	5'CCCTTTTTGCTGCTAGTATCC 3'	112 – 132 NM_000493
antisense	5'CTGTTGTCCAGGTTTTCCTGGCAC 3'	556 – 579 NM_000493

Collagen type XI		
sense	5'ACTTCTGACTGCCTCTGCTC 3'	5418 – 5437 NM_001854
antisense	5'GCTTTTGCCATGTGATTCTGCC 3'	5891 – 5912 NM_001854

Aggrecan		
sense	5'GCCTTGAGCAGTTCACCTTC 3'	1814 – 1833 NM_001135
antisense	5'CTCTTCTACGGGGACAGCAG 3'	2186 – 2205 NM_001135
Chondroadherin		
sense	5'ACCTGGACCACAACAAGGTC 3'	535 – 554 NM_001267
antisense	5'CACCTTCTCCAGGTTGGTGT 3'	907 – 923 NM_001267

Fibromodulin		
sense	5'CTTACCCCTATGGGGTGGAT 3'	175 – 194 NM_002023
antisense	5'AAGTAGCTATCGGGGACGGT 3'	792 – 811 NM_002023

### Immunohistochemistry

MSC undergoing osteogenic differentiation were subjected to immunohistochemical analysis for CYR61 expression. Cells were rinsed with PBS, covered with a glass cover slip and analyzed immediately. As the primary antibody an anti mouse CYR61 polyclonal antiserum (Munin, Chicago, USA) at a 1:100 dilution in PBS was used and the slides were incubated at 4°C overnight. The further steps were carried out at room temperature. Following three 5 min washes with TBS a monoclonal mouse anti rabbit IgG (DAKO, Hamburg, Germany) antibody at a 1:50 dilution in a solution consisting of 100 μl human AB plasma in 700 μl PBS was added and incubated for 30 min. After rinsing with PBS a rabbit anti mouse IgG antiserum (DAKO, Hamburg, Germany) diluted 1:25 was added and incubated for 10 min. After rinsing in PBS cells were incubated with a complex of intestinal alkaline phosphatase and mouse monoclonal anti alkaline phosphatase antiserum (APAAP) (DAKO, Hamburg, Germany) at a 1:50 dilution for 10 min. The final signal intensity was amplified using 2 additional cycles of incubations with the rabbit anti mouse IgG antiserum and the APAAP complex. Signals were developed using a conventional freshly prepared fast-red staining solution (40 mg fast red, 18 mg levamisole and 20 mg naphtol-AS-MX-phosphate, 1 ml DMF and 40 ml propanediol-buffer consisting of 50 mM 2-amino-2 methyl 1,3 propanediol pH = 8.7). Following a 10 min incubation, sections were washed in ddH_2_O and prepared for microscopy.

During every analysis a negative control was performed according to Wong et al. [22]. Sections were incubated using a rabbit serum instead of the primary antibody (rabbit anti CYR61 antiserum) at a similar protein concentration. Microscopic images were acquired using a digital camera and IP-Lab-Spectrum analysis software.

Immunohistochemical analyses for markers of chondrogenic differentiation were performed using the antisera. Immunoreactivity was detected using a streptavidin-peroxidase staining as described [36].

## Competing interests

The author(s) declare that they have no competing interests.

## Authors' contributions

NS designed and interpreted the experiments, as well prepared the manuscript. UN and CH selected the patients for MSC cell culture, JS isolated the MSC and FJ provided overall guidance.
